# Different frequencies of human scalp-recorded theta activity may index integration of activity in distinct recurrent cortico-subcortical mnemonic networks

**DOI:** 10.3389/fnbeh.2025.1686252

**Published:** 2025-10-15

**Authors:** Inanna K. Haddon, Rohan O. C. King, Dylan A. Taylor, Jodie N. Bell, Jasmine E. B. Murray, Meghan van der Meer, Christopher D. Erb, Ian J. Kirk

**Affiliations:** School of Psychology and Centre for Brain Research, University of Auckland, Auckland, New Zealand

**Keywords:** frontal–parietal and fronto-temporal theta oscillations, theta-gamma coupling, frontal midline theta, memory, Alzheimer’s disease

## Abstract

It is now well-accepted that differing frequencies of neuro-oscillations support the selection, quantising, and pacing of information around different circuits in the brain. Another related function of neuro-oscillations, for which the frequency of oscillation is again critical, is to allow for integration of neural activity across differing spatial scales. In this short review, we discuss the degree to which human scalp-recorded EEG occurring in the theta-range (4-8 Hz) can be used to infer activation of mnemonic circuits involving the hippocamps and diencephalon (Papez loops), as well as in the neocortical areas the activity is directly recorded from. We also discuss the potential role of theta-range frequency modulation in the selection of specific mnemonic circuits. In light of the foregoing, we suggest that the frequency at which theta is occurring within and between cognitive tasks should be reported more thoroughly than it generally is. Finally, we suggest that assessing disruptions in frequency modulation of theta-range oscillations is a potentially valuable biomarker for disorders such as Alzheimer’s disease.

## Introduction

**Figure 1:**
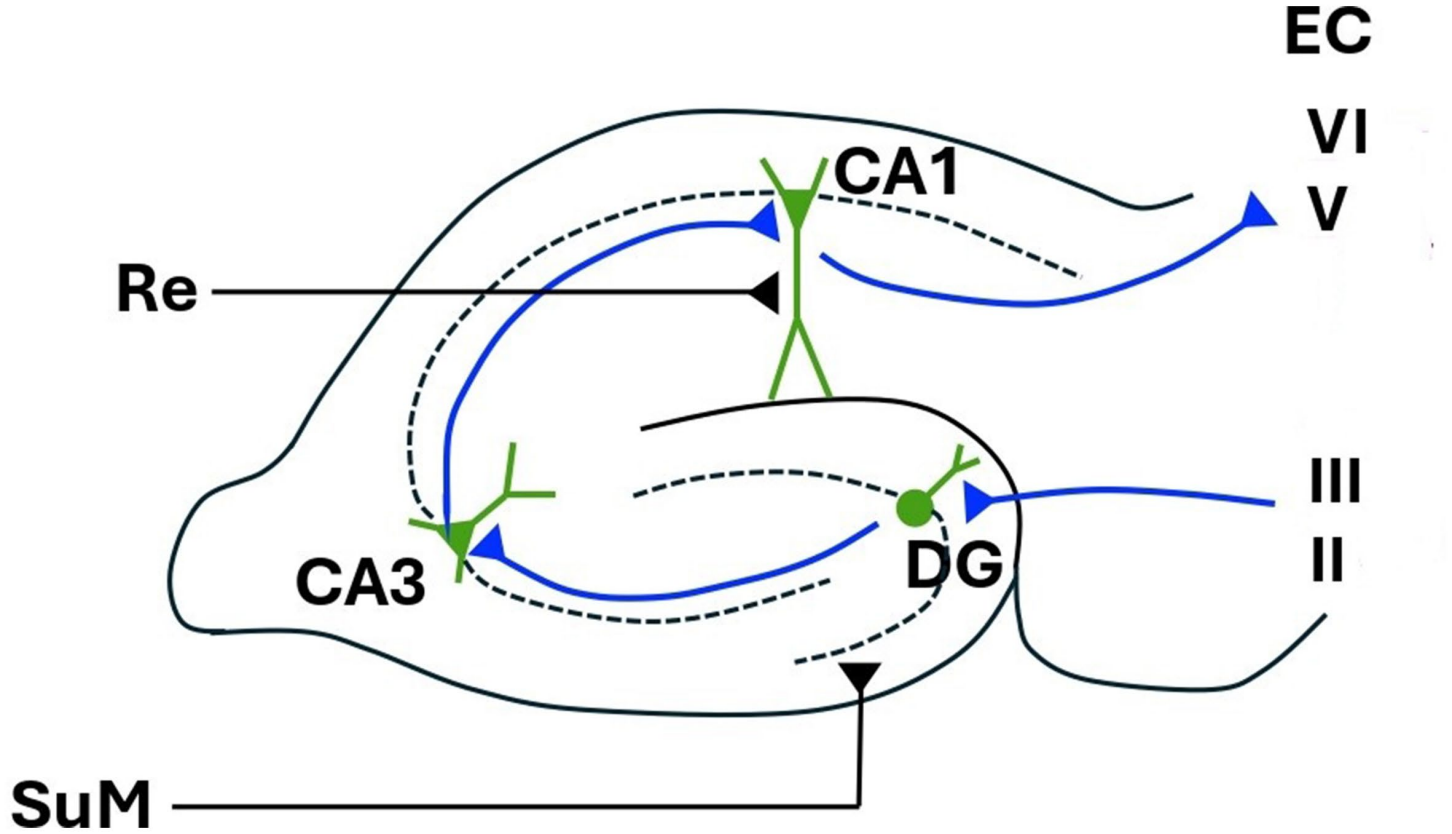
Hippocampal tri-synaptic loop. EC, entorhinal cortex; DG, dentate gyrus; CA1 and CA3, cornu ammonis 1 and 3; Re, nucleus reuniens; SuM, supramammillary nucleus. Figure modified from López-Madrona et al. (2017).

The intracranially recorded hippocampal EEG (usually obtained from animals) is characterised by a large-amplitude, pseudo-sinusoidal waveform known as theta rhythm (O’Keefe and Nadel, 1978; Miller, 1991; Gray and McNaughton, 2000; Mitchell et al., 2008). Due to the rhythmic nature of theta, and that its frequency is finely modulated [see Kirk (1998)], it is usually assigned a role in timing or quantising the passage of information around various hippocampal-related circuits. For example, it has been suggested that one function of theta is to organise information flow through the hippocampal tri-synaptic loop and associated structures. That is, from entorhinal cortex → dentate gyrus → CA3 → CA1 → subiculum; and/or around a subiculum → lateral septum → medial septum → hippocampus (O’Keefe and Nadel, 1978; Gray and McNaughton, 2000; Stepan et al., 2015; Farrell and Soltesz, 2025; see Figure 1). An equivalent role of theta has also been suggested for more spatially distributed loops. Theta (and the precise frequency of theta) might allow selection from a variety of potential recurrent loops (originally proposed by Papez, 1937) comprised of nuclei in the anterior thalamic complex, the mamillary body, and hippocampus (Parmeggiani et al., 1971, 1974; Gray and McNaughton, 2000).

Theta-range EEG activity is also readily recorded from the scalp electrodes in human participants. It is most prominent over the frontal-midline, and has hence become known as frontal-midline theta (Mitchell et al., 2008). However, theta-rhythmic oscillations can be recorded across wide regions of the neocortex (von Stein and Sarnthein, 2000; Kirk and Mackay, 2003; Mitchell et al., 2008; Kawasaki et al., 2014). Indeed, Miller (1991) suggested that theta acts to establish a rhythmic interplay (resonance) between the hippocampus and various neocortical regions. Recurrent loops between the hippocampus and different neocortical areas will have different path lengths, and thus different circuits, with different return times will be preferentially selected by different frequencies of theta. That is, a particular frequency of theta will select a particular neocortical component of the circuit to be entrained into resonant activity with the hippocampus.

It has also been argued that different frequencies of oscillation bind or integrate neocortical areas across different spatial scales (von stein and Sarnthein, 2000). High-frequency gamma-band oscillations might bind neighbouring neural nodes, within the visual cortex for example, via short interconnections. Lower frequency oscillations within the theta-band might co-ordinate activity across greater spatial scales, with longer range interconnections. It follows therefore that within the theta-range, different frequencies might integrate different neocortical networks.

In the following brief review, we discuss the degree to which human scalp-recorded EEG occurring in the theta-range (4-8 Hz) might be used to infer activation and integration across different nodes of the circuitry of the kind discussed above. The first (and simplest) case we will consider is that of theta occurring across the neocortex. We will consider whether we might expect different frequencies of theta to bind fronto-parietal vs. fronto-temporal networks, for example. As the subcortical (e.g., Papez) circuits discussed above include potential neocortical components, we will also discuss the possibility that scalp-recorded theta-range neocortical oscillations might also provide a window into the state of these more distributed circuits, even though we are unable to record electrical activity directly from the hippocampal and/or diencephalic components of these circuits.

It is worth noting here too that hippocampal theta, and indeed hippocampal function in general, usually focusses on a well-established involvement in mnemonic processes (O’Keefe and Nadel, 1978; Mitchell et al., 2008; Colgin, 2013; Buzsàki and Moser, 2013; Lisman and Jensen, 2013; Korotkova et al., 2018; Karakas, 2020). However, hippocampal theta likely also occurs in other non-mnemonic processes (Bland and Oddie, 2001; Pan and McNaughton, 2004; Korotkova et al., 2018; Karakas, 2020), and it has been suggested that neocortical frontal midline theta might be a signature of cognitive control (Cavanagh and Frank, 2014), and thus occurs during mnemonic processes (Sauseng et al., 2005; Berger and Sauseng, 2022), but is not limited to them. Here, however, we will largely limit ourselves to the discussion of theta in mnemonic processes, and the potential disruptions that may accompany memory loss in disorders such as Alzheimer’s.

### Fronto-midline theta, frontal–parietal and fronto-temporal theta oscillations, and theta-gamma coupling

As noted above, one role of oscillations in general is to integrate neocortical areas across different spatial scales (von stein and Sarnthein, 2000). It follows therefore that different frequencies within the theta-range might differentially engage different neocortical networks in different cognitive tasks. Working memory involves the short-term selection, maintenance, and manipulation of memory information (Baddeley, 1986, 2003), and increased theta power over frontal regions has consistently been reported in human EEG (and MEG) during working memory tasks (Mitchell et al., 2008; Klimesch, 1999; Jensen and Tesche, 2002; Hsieh and Ranganath, 2014). The power of frontal-midline theta also increases with working memory load (Klimesch, 1999; Meltzer et al., 2007, 2008; Scheeringa et al., 2008; Hsieh et al., 2011; Roberts et al., 2013).

Of particular note here, is the repeated observation of frontal midline theta coherent with additional theta-range EEG oscillations over the temporal (Anderson et al., 2010; Kawasaki et al., 2010; Kawasaki et al., 2014) or parietal lobes (Kawasaki et al., 2010; Kawasaki et al., 2014; Griesmayr et al., 2014; Berger et al., 2019) in, respectively, verbal or visuospatial working memory. On the argument we present here, coherent theta-range oscillations over the frontal and temporal lobes are indicative of the selection of a frontal-temporal network in verbal working memory tasks, while coherent theta-range oscillations in frontal and parietal regions indicates the selection of a frontal–parietal network for spatial working memory tasks. Further, we suggest that the frequency at which theta occurs will be significantly different in verbal versus spatial tasks.

In a range of studies in Pavlov and Kotchoubey (2022) for example, it appears that frontal-midline theta in verbal tasks may occur at higher frequency than in visuo-spatial tasks. Unfortunately, our confidence in this observation is limited. Most importantly, most published studies report a range of theta rather than precise frequencies, requiring estimates to be made from published figures. It is also likely that there will be a degree of individual variability in the frequency (or range of frequencies) of theta-band activity as there is for alpha [see for, e.g., Klimesch (1997)], and working memory studies employ a variety of different designs even when studying ostensibly the same cognitive task (Pavlov and Kotchoubey, 2022). Thus, cross-study comparisons are not ideal. Indeed, more repeated measures designs, in which the same subjects are tested in two or more memory tasks (Kawasaki et al., 2014), in which the frequency of theta is specifically measured, are needed.

In addition to the study of frontal midline theta per se, there is also considerable work assessing the role of gamma oscillations nested in theta (or cross-frequency coupling). It has been suggested, for example, that separate items of information are held on different sub-cycles of gamma allowing for information to be stored in a temporally sequenced manner [see Lisman and Idiart (1995), Jensen and Colgin (2007), Lisman and Jensen (2013), and Sheremet and Qin (2025)]. On this argument, the number of gamma sub-cycles nested onto a single theta-cycle determines the number of items that can be stored in working memory. It follows therefore that alterations in the frequency (and therefore wavelength) of theta will affect storage capacity. Theta-gamma coupling has been demonstrated in the human hippocampus (Canolty et al., 2006; Axmacher et al., 2010; Daume et al., 2024) and has been repeatedly demonstrated across frontal–parietal networks in human EEG during working memory tasks (Sauseng et al., 2009; Berger et al., 2019). Further, and specific to the current discussion, manipulations of theta frequency (and thus wavelength) have been shown to acutely alter working memory capacity in the hypothesised direction – that is, lowering the frequency of theta increased the number of items that are stored in working memory (Wolinski et al., 2018; Akturk et al., 2022).

Theta-gamma coupling has also been used to assess working memory in the elderly (Abubaker et al., 2024), and in people with mild cognitive impairment (MCI) or Alzheimer’s disease (AD; Goodman et al., 2018). MCI is considered the prodromal phase of AD, and prefrontal function in MCI has been used to predict progression to AD (Gomar et al., 2011). Goodman et al. (2018) found that AD patients had the lowest level of theta-gamma coupling, followed by MCI and then control participants. Theta-gamma coupling was also found to be the most significant predictor of working memory performance.

Theta-gamma coupling and working memory might therefore serve as an indicator (or biomarker) for those likely to progress from MCI to AD. Theta-gamma coupling might also serve as an assay for the efficacy of therapeutic interventions, or may be a target for them. For instance, Diedrich et al. (2025) applied tACS to dorsolateral prefrontal cortex and showed some improvement in a working memory task in elderly participants. Interventions of this sort may well prove to be of benefit in AD or MCI populations.

### Hippocampal theta and cortico-hippocampal interplay

Although neocortical networks may be sufficient to maintain information in working memory in some tasks (Nyberg and Eriksson, 2016), it is likely that most longer-term mnemonic tasks – targeting episodic, recognition, or working memory – require hippocampal involvement (Kirk and Mackay, 2003; Mitchell et al., 2008; Cashdollar et al., 2009; Leszczynski, 2011; Yonelinas et al., 2023). As noted above, theta activity occurring across cortical-hippocampal loops is proposed to co-ordinate this involvement (Miller, 1991). Further, Miller proposed that different frequencies of hippocampal theta would select different cortico-hippocampal loops.

The ascending system that modulates hippocampal theta frequency has been reviewed extensively previously (Kirk, 1998; Vertes and Kocsis, 1997; Kirk and Mackay, 2003; Pan and McNaughton, 2004). To briefly summarise, hippocampal theta is possibly driven by rhythmically-bursting pacemaker cells in the medial septum/diagonal band of Broca or, as some modelling studies suggest, theta may be an intrinsic property of a septo-hippocampal reciprocal loop (Denham and Borisyuk, 2000; Wang, 2002). Either way, ascending pacemaker activity from theta bursting cells in the supramammillary nucleus (SuM) of the hypothalamus provides a theta-rhythmic pacemaker signal to the medial septum, that in turn determines the frequency of hippocampal theta. In turn, reciprocal descending input to SuM from the septo-hippocampal system modulates SuM discharge frequency, thereby maintaining fine control of theta frequency. It should be noted, however, that SuM may only determine theta frequency during some behaviours. During other behaviours theta in the septo-hippocamapal system may be independent of SuM input (Kirk, 1998; Denham and Borisyuk, 2000; Kirk and Mackay, 2003; Pan and McNaughton, 2004).

In any case, different frequencies of hippocampal theta produced by different behaviours will produce different cortico-hippocampal loops. Again, this should lead to different topographies of neocortical theta, occurring at different frequencies, for different behaviours. It is generally assumed that coherent theta in neocortex and hippocampus described here is due to interaction via the entorhinal cortex (EC), and certainly this is the bi-directional pathway proposed by Miller (1991) for theta-modulated cortico-hippocampal resonant loops. In freely moving animals however, Chrobak and Buzsàki (1994) found theta modulated activity in cells of the input layers of the EC (i.e., in the EC relay from neocortex to hippocampus, layers I-III, see Figure 1), but not in the cells of the output layers (i.e., in the EC relay from neocortex to hippocampus, layers I-V-VI; see Figure 1). Thus, neocortically recorded theta (including frontal midline theta), if coherent with ongoing hippocampal theta, may indicate that a neocortical to hippocampal input pathway is currently active. An MEG study showing that theta from the pre-frontal cortex drove that of the hippocampus during performance of a mismatch task (Garrido et al., 2015) is consistent with this view. As cells in the hippocampal output layers of the EC (Chrobak and Buzsàki, 1994; see Figure 1) are not theta modulated in freely moving animals, the hippocampal to cortex part of a recurrent cortico-hippocampal loop proposed by Miller (1991) has been suggested to be not via the EC (Kirk and Mackay, 2003; Mitchell et al., 2008). Kirk and Mackay (2003) also note that the theta-modulated re-entrant loop might be completed via descending projections to the medial mammillary bodies, and back to neocortex via the anterior thalamic complex (see section 4 below). Observations of hippocampal theta leading that of frontal theta (Siapas et al., 2005) are perhaps consistent with activation of this circuit. Of note however, recordings from intracranial electrodes during a working memory task in humans suggest that theta/alpha band activity co-ordinates unidirectional communication from hippocampus to EC (Li et al., 2024). It is perhaps likely that theta modulated flow through the hippocampus and associated circuitry is very much task dependent.

The SuM, as well as projecting to and providing theta-rhythmic input to the medial septum, also directly projects to the dentate gyrus and CA2 of the hippocampus. Another ascending projection from the nucleus reuniens of the thalamus (Re) provides complimentary input to the CA1 and subiculum (Vertes, 2015). The SuM and Re may, respectively, modulate activity in the neocortical to hippocampal input pathway, and the hippocampal to subcortical output pathway discussed above. The Re is also reciprocally connected to the prefrontal cortex and may relay information between hippocampus and frontal cortex (Vertes et al., 2007; Ito et al., 2018). Volume reductions in Re have recently been shown to be a potential biomarker for progression to Alzheimer’s (Censi et al., 2024). Dysfunction of the Re-hippocampal pathway might specifically affect theta-modulated output from the hippocampus. Thus, there exists the possibility of human scalp recorded theta being used to assess relative integrity of hippocampal output (relative to input) pathways, and thus Re function.

### Theta oscillations and re-entrant loops in Papez circuit

**Figure 2:**
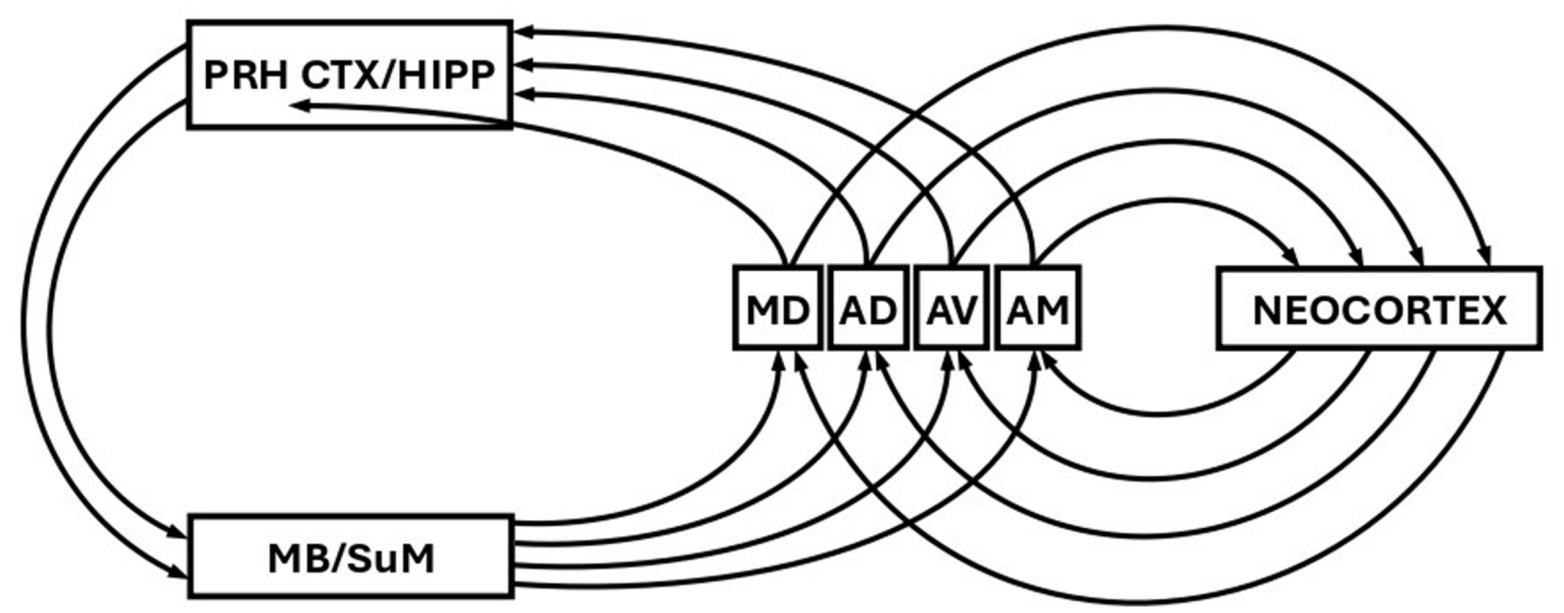
Schematic showing the re-entrant loops of the Papez circuit. PRH CTX, perirhinal cortex; HIPP, hippocampus; MB, mammillary bodies; SuM, supramammillary nucleus; MD, mediodorsal thalamic nucleus; AD, anterodorsal thalamic nucleus; AV anteroventral thalamic nucleus; AM, anteromedial thalamic nucleus. Figure modified from Parmeggiani et al. (1974).

Finally, and consistent with the theme developed so far, it was suggested over fifty years ago (Parmeggiani et al., 1971, 1974) that a function of theta might be to control the selection of re-entrant loops around circuits originally proposed by Papez (1937); see Figure 2. This general idea has been revisited and refined many times since (e.g., Kirk, 1998; Gray and McNaughton, 2000; Kirk and Mackay, 2003; Dalrymple-Alford et al., 2015; Perry and Mitchell, 2019; Aggleton et al., 2022; McNaughton and Vann, 2022). Consistent with this idea, however is that theta-rhythmic activity has been found in mammillary nuclei (Kocsis and Vertes, 1994; Bland et al., 1995; Kirk et al., 1996), and in the anteroventral (AV) anteromedial (AM) and anterodorsal (AD) nuclei (Kirk et al., 1997; Vertes et al., 2001) in addition to that of the septohippocampal system and SuM discussed above. As discussed previously (Kirk, 1998; Kirk and Mackay, 2003), theta activity in mammillary bodies is likely driven by descending input from the septo-hippocampal system, and mammillary bodies subsequently drive the anterior thalamic nuclei (AV, AM, and AD). The anterior thalamic nuclei (AT) project back to the hippocampal region (and to neocortex), thus completing Papez circuit (or a more recent interpretation of it). Theta-rhythmic activity has also been reported in mediodorsal thalamus (MD; Kirk et al., 1997), but it is not in receipt of mamillary body input, and is thus not part of the same parallel re-entrant circuitry as AV, AD, and AM. However, the MD is in receipt of afferent input from Sum, and is therefore in receipt of theta frequency input (Kirk and Mackay, 2003). Thus MD, as outlined below, might still be part of a parallel mnemonic re-entrant system.

The anterior thalamic nuclei also reciprocally connect to neocortex (Shibata, 1993a, 1993b; Mathiasen et al., 2020), forming additional re-entrant loops (Figure 2). If different frequencies of theta select for different re-entrant loops involving different anterior thalamic nuclei, then this will likely be reflected in different frequencies of theta recorded from neocortex. Again, there is therefore the possibility we might assess deep activation via scalp recorded EEG in humans. Activation in different deep Papez loops involving the anterior thalamic nuclei may reflect different stages or depth of processing (McNaughton and Vann, 2022). The MD is also reciprocally connected to the neocortex (Mitchell, 2015) and thus the same argument applies albeit for a somewhat different circuit. Of particular relevance here is the argument that two separate re-entrant loops may be involved in two distinct processes of recognition memory. It has been argued that a process of recollection might involve a circuit that includes hippocampus and AT, while familiarity judgements involve a circuit that includes perirhinal cortex and MD (Aggleton and Brown, 1999). Again, if theta frequency is involved in the selection of these two processes, this should be reflected in different frequencies of scalp recorded theta generated in the theta-modulated targets of AT and MD.

Finally, and again with respect to clinical applications, perirhinal cortex might be amongst the first brain areas affected in the early stages of Alzheimer’s (Hirni et al., 2016), and there is some evidence that familiarity-based memory deficits might be a specific behavioural marker for Alzheimer’s (e.g., Wolk et al., 2013). This further motivates investigation of scalp-recorded theta-range oscillations in the EEG occurring during familiarity tasks (as opposed to recollection tasks) as a potential biomarker for the early and/or prodromal stages of Alzheimer’s.
